# Risk management patterns in radiation oncology—results of a national survey within the framework of the Patient Safety in German Radiation Oncology (PaSaGeRO) project

**DOI:** 10.1007/s00066-022-01984-5

**Published:** 2022-08-05

**Authors:** Andrea Baehr, Daniel Hummel, Tobias Gauer, Michael Oertel, Christopher Kittel, Anastassia Löser, Manuel Todorovic, Cordula Petersen, Andreas Krüll, Markus Buchgeister

**Affiliations:** 1 Outpatient Center of the UKE GmbH, Department of Radiotherapy and Radiation Oncology, University Medical Center Hamburg-Eppendorf, Martinistr. 52, 20251 Hamburg, Germany; 2 Department of Radiotherapy and Genetics, Outpatient Center Stuttgart, University Hospital Tübingen, Stuttgart, Germany; 3 Department of Radiotherapy and Radiation Oncology, University Medical Center Hamburg-Eppendorf, Hamburg, Germany; 4 Department of Radiation Oncology, University Hospital Münster, Münster, Germany; 5 Faculty of Mathematics—Physics—Chemistry (II), Berliner Hochschule für Technik, Berlin, Germany

**Keywords:** Risk analysis, Radiotherapy, Quality management, Failure culture, Failure modes and effects analysis (FMEA)

## Abstract

**Purpose:**

Risk management (RM) is a key component of patient safety in radiation oncology (RO). We investigated current approaches on RM in German RO within the framework of the Patient Safety in German Radiation Oncology (PaSaGeRO) project. Aim was not only to evaluate a status quo of RM purposes but furthermore to discover challenges for sustainable RM that should be addressed in future research and recommendations.

**Methods:**

An online survey was conducted from June to August 2021, consisting of 18 items on prospective and reactive RM, protagonists of RM, and self-assessment concerning RM. The survey was designed using LimeSurvey and invitations were sent by e‑mail. Answers were requested once per institution.

**Results:**

In all, 48 completed questionnaires from university hospitals, general and non-academic hospitals, and private practices were received and considered for evaluation. Prospective and reactive RM was commonly conducted within interprofessional teams; 88% of all institutions performed prospective risk analyses. Most institutions (71%) reported incidents or near-events using multiple reporting systems. Results were presented to the team in 71% for prospective analyses and 85% for analyses of incidents. Risk conferences take place in 46% of institutions. 42% nominated a manager/committee for RM. Knowledge concerning RM was mostly rated “satisfying” (44%). However, 65% of all institutions require more information about RM by professional societies.

**Conclusion:**

Our results revealed heterogeneous patterns of RM in RO departments, although most departments adhered to common recommendations. Identified mismatches between recommendations and implementation of RM provide baseline data for future research and support definition of teaching content.

**Supplementary Information:**

The online version of this article (10.1007/s00066-022-01984-5) contains supplementary material, which is available to authorized users.

## Introduction

Radiation oncology (RO) plays a vital role in cancer management with almost half of all patients undergoing radiation therapy (RT) during their course of disease [1]. Modern RT techniques complement patient-individualized treatment planning and image-guided dose delivery by adaptive radiation therapy from daily imaging and patient repositioning to daily treatment planning. With modern techniques, even small dosimetric deviations can result in significant errors, leading to a devastating impact in terms of side-effects and therapy success [2–5]. In recent years, several organizations have focused on patient safety during RT, providing recommendations for radiation oncology professionals [3, 6–8].

Several publications reported about occurrence of incidents during RT, e.g., application of a wrong plan, mistaken patient identity, underdosage on tumor tissues, or ignorance of previously applied dosages [3, 9, 10]. The rate of incidents leading to major or long-lasting harm have decreased during the last few decades; nevertheless, newer data suggest that about 1% of incidents contribute to patients’ premature death [2]. For Germany, numbers of incidents which fulfill certain conditions of severity as reported to a public authority reporting system are available, i.e., 49 RT incidents were reported nationwide in 2020 [11]. As only highly dangerous incidents are reported here, the number of incidence actually occurring during daily treatment might be much higher [12]. Risk management now offers the opportunity to anticipate possible failures, systematically learn from incidents, and implement measures to reduce risks which contributes to a safe therapy environment and is recommended by several authorities [8, 13–15].

Prospective RM is one key component of RM and is used to evaluate processes in patient care in terms of possible pitfalls, e.g., mistake of identities [16]. It is recommended to perform these analyses with an interprofessional team before implementation of new processes or routinely, e.g., annually [6, 7, 16]. Topic of analysis might be only one step of the treatment process (subprocess) or even several processes altogether [16]. Several methods might be of help and especially failure modes and effects analysis (FMEA) with nomination of values to rank risks or use of a risk matrix are the topic of several publications [16–18]. Based on this analysis, measures can be initiated and implemented in daily routine to minimize the occurrence or the severity of errors. Reactive RM or analysis of adverse events is another key component of RM. It consists of reporting and processing of incidents and the subsequent definition of measures [2]. Multiple systems to report incidents are established and range from international systems, e.g., ROSEIS (“radiation oncology reporting and education system” of the European Society for Radiation Oncology [ESTRO]) to in-house solutions for the department [19, 20]. What is learned from incidents and near-events (meaning failures which did not reach the patient) is valuable knowledge and should be processed preferably in an interprofessional team and translated into implementation of safety barriers [21–23]. Open communication about risk analyses and reported incidents support built-up of a sustainable safety culture and is highly recommended [15, 23]. Common methods to establish RM include conducting morbidity and mortality conferences (m&m conferences) [24] or implementing a safety assurance/RM manager and/or committee [22, 23]. Accreditations of institutional quality management (QM) are a common tool to evaluate QM and in RM (e.g., according to DIN EN 15224) in order to provide high-quality care [15, 23, 25]; however, the role of accreditation regarding patient safety has to be evaluated further [26]. To underline the importance of a systematic risk management for German health care processes, it was declared to be mandatory by the patients’ right act [27] for health care in general and by the new law on radiation protection ordinance and consecutive legislation for RT in 2018 [28] which translates the EU legislation on the national level [29].

From 2011, the ACCIRAD project analyzed Europe-wide approaches on implementation of RM tools in RO [30]. The authors evaluated national legislation and recommendations on prospective and reactive risk management. Several countries having implemented different national regularities were identified, whereas no such implementation in national modus operandi for Germany was reported at that time. Multiple guidelines from different international societies and papers about best practice in RM in RO have been published [6, 7, 17, 31, 32]. These offer recommendations on different dimensions of RM as described in the above section and promote growing knowledge on that topic in RO society. To complement these insights, we conducted a nationwide survey within the Patient Safety in German Radiation Oncology (PaSaGeRO) project to evaluate the current modus operandi for RM in Germany. PaSaGeRO is a joint project between the German Society for Medical Physics (DGMP) and the German Society for Radiation Oncology (DEGRO), investigating current approaches concerning patient safety and to define key components for a sustainable safety culture in German RO. In the past, interprofessional discussion rounds, e.g., in the context of meetings of the DGMP working group on risk management demonstrated insecurity and discordance among radiation oncology experts concerning implementation of risk management methods, protagonists or tools. Furthermore, these discussions underlined the strong relevance of risk management for radiation oncology processes not only to fulfill legal requirements, but to ensure safety during patients’ paths in therapy. Therefore, aims of this evaluation are to:

- Present the status quo of the patterns and needs of risk management in Germany. Hereby, we aim to evaluate the fulfillment of recommendations and regulations as described in the introduction. The results offer the possibility to create specific continuous-education courses or recommendations. Particularly addressed can be processes, tools, or topics of risk management which might differ from international recommendations or legal requirements and implementation in clinical routine.
- Enhance communication about risk management in general and subtopics such as incident reporting in particular. Systematic elaboration of the results offers the possibility for all German radiation oncology experts to compare our own approaches with current approaches in other departments and to advance the in-house risk management processes.
- Build a base for future research on questions of risk management or patient safety in general in RO, e.g., to investigate differences in approaches for RM in different institutions, methods for facilitated implementation of RM tools, or assessment of existing approaches.

This represents the first systematic evaluation of RM approaches in German RO. The results can contribute knowledge to develop new recommendations and teaching content based on national legislation and existing guidelines regarding modern RO strategies.

## Materials and methods

We developed a questionnaire to address the following aspects: prospective RM, reactive RM, protagonists of RM, self-assessment concerning RM, and general data on the participating RO department. Items were designed to cover the above-mentioned components of risk management as found in national and international recommendations. It was designed using LimeSurvey (LimeSurvey GmbH, Hamburg, Germany) containing 18 questions in multiple-choice style. Self-assessment concerning knowledge on RM was answered using a 5-point Likert scale: very good (1)–good (2)–satisfying (3)–bad (4)–very bad (5). Process steps as mentioned in the questionnaire were aligned to the WHO radiotherapy risk profile [3]. The set of subprocesses was adapted and finalized after several interprofessional discussion rounds with the aim to keep it well arranged and suitable for several institutional-specific workflows. The full questionnaire is available as supplementary material as an English transcript. An invitation link was first sent via e‑mail trough the official DGMP mailing list, second to a mailing list for medical physicists in Germany which is also open for non-DGMP members, and third also to all university hospital RO department in Germany, to reach possibly all (*n* = 294 [33]) German RO departments. The survey was available between June and August 2021. Institutions were asked to answer the questions once per institution by a physicist, physician, or other member of the team. All answers were returned anonymously. All participants provided consent to data publication. Data were analyzed using SPSS statistics 28 (IBM, Armonk, NY, USA). Normal distribution was tested using the Kolmogorov–Smirnov test. Categorial data were compared via χ^2^ test and Fisher’s exact test. The Kruskal–Wallis test was used to compare median values of different groups. A *p*-value of ≤ 0.05 was defined to be significant.

## Results

In all, 48 completed forms were eligible for evaluation: 17 institutions were university hospitals (UH; 35%), 11 were general and non-academic hospitals (23%), and 16 (33.3%) were private practices/medical service centers. Four participants did not name their affiliation.

At that time, 21 institutions (44%) had an official accreditation for their QM (e.g., according to DIN EN 15224:2015), of which most were UH (*n* = 12, 71% of all UH). Other hospitals and practices infrequently reported an accreditation (4/11 [36%] and 5/16 [31%], respectively). Significant differences were seen in the comparison of UH and private practices (*p* = 0.038).

**Fig. 1:**
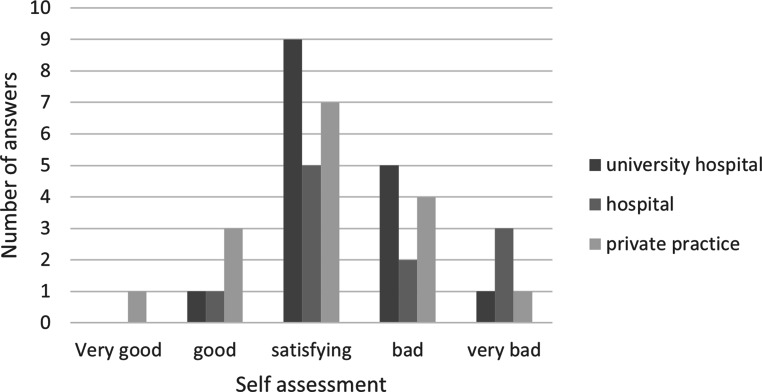
Self-assessment concerning knowledge on risk management (RM) as specified by participating institutions. Answers according to type of institution

Most institutions rated their knowledge concerning risk management as “satisfying” (21, 44%) or “bad” (11, 23%). Median value was three (“satisfying”) for all ratings. No difference in median value of UH vs. hospital vs. practice was seen (*p* = 0.34). Sixteen departments ranked their knowledge to be bad or very bad (33%; Fig. 1). No significant difference between (university) hospitals and practices was seen (*p* = 0.54). No difference between departments with or without accreditations were seen (*p* = 0.78).

**Fig. 2:**
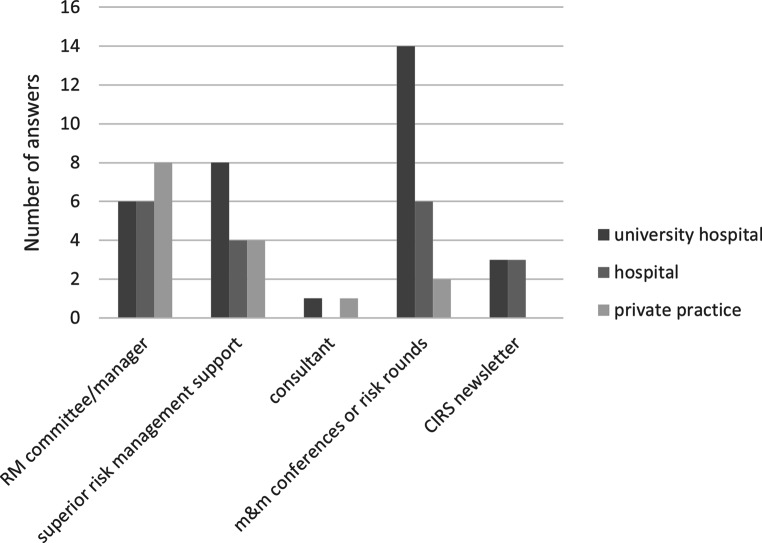
Number of risk management (RM) tools and protagonists as reported by participants broken down for different types of institutions. *m&m* morbidity and mortality, *CIRS* critical incident reporting system

**Fig. 3:**
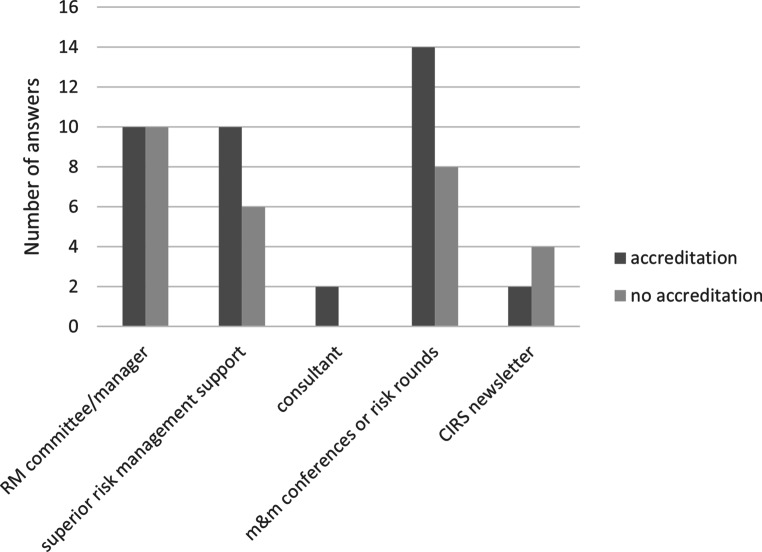
Number of risk management (RM) tools and protagonists as reported by participants broken down for the status of accreditation for quality management (QM)* m&m* morbidity and mortality, *CIRS* critical incident reporting system

Risk rounds or m&m conferences were implemented in 22 departments (46%). Twenty (42%) departments had an explicit manager or committee for RM, while 16 (33%) had a dedicated specialist from the superior hospital risk management. Two institutions reported about external consultants for risk management purposes. Accredited departments rather tended to have risk rounds/m&m conferences (*p* = 0.069), so did UH (*p* = 0.04), Figs. 2 and 3.

A total of 31 participants (65%) wished for more information on RM through their respective society, 30 participants (63%) would prefer information to be provided via special courses on RM in RO, and 8 (17%) would prefer more information on the topic during university education.

## Prospective risk management

In all, 42 institutions had undertaken at least one risk analysis so far (88%), of which 23 reported multiple or regular analyses (e.g., yearly, 48%). Five departments had not yet conduct prospective risk analyses (10%); one department did not answer this question. Departments with a risk management committee or risk manager reported significantly more often on risk analyses performed at multiple occasions or on a regular basis (*p* < 0.01). Type of institution or achievement of accreditation did not impact number of analyses being performed.

In 31 (65%) institutions, analyses were performed with an interprofessional team in multiple conferences; in 17 (35%), only one professional group was responsible, of which medical physics was in charge in 13 cases (76%). No difference between different types of departments was seen in this aspect. The most common method for prospective risk analyses was failure modes and effects analysis (FMEA, used by 25 institutions, 52%). Use of risk matrix or fault tree analyses was less common, while 6 (13%) participants used a combination of different methods.

Furthermore, 44 institutions reported information on subprocesses they included in the analyses. The number of subprocesses included ranged between 1 and 12 of 13 suggested steps. Most commonly imaging, planning, patient setup, and first treatment were analyzed. One institution reported on analysis of possible errors during chemo-/immunotherapy. Average total number of subprocesses being included into analyses were higher in departments with risk committee or risk manager (8.9 vs. 7.1) and departments with accreditation of QM (8.8 vs 7.1). No difference between type of institution was seen. Results for respective subprocesses are provided in Table 1.

**Table 1:** Subprocesses as analyzed in the prospective risk analyses. Columns showing total number/percent of institutions having included the respective subprocess stratified in three categories

Subprocess		RM committee/manager			Accreditation			Institution			
(total number)	All institutions (44)	Yes (20)	No (24)	*p*	Yes (21)	No (23)	*p*	University hospital (17)	Hospital (11)	Private practice (16)	*p*
Patients’ assessment	28 (63%)	16 (80%)	12 (50%)	0.04	18 (86%)	10 (43%)	< 0.01	10 (58%)	7 (64%)	11 (69%)	0.8
Decision to treat and prescription	28 (63%)	17 (85%)	11 (46%)	< 0.01	16 (76%)	12 (53%)	0.1	11 (65%)	8 (73%)	9 (56%)	0.68
Positioning	33 (75%)	16 (80%)	17 (71%)	0.84	17 (81%)	16 (69.5%)	0.38	14 (82%)	7 (64%)	12 (75%)	0.5
Imaging	34 (77%)	16 (80%)	18 (75%)	0.69	18 (85.7%)	16 (69.5%)	0.2	15 (88%)	7 (64%)	12 (75%)	0.3
Volume definition	24 (55%)	13 (65%)	11 (46%)	0.2	15 (71%)	9 (39%)	0.03	12 (70.5%)	3 (27%)	9 (56%)	0.08
Data transfer	21 (48%)	9 (45%)	12 (50%)	0.74	11 (52%)	10 (43%)	0.9	10 (58%)	5 (45%)	6 (37.5%)	0.4
Planning	35 (79.5%)	17 (85%)	18 (75%)	0.4	19 (90%)	16 (69.5%)	0.08	15 (88%)	8 (73%)	12 (75%)	0.5
Patient setup and first treatment	37 (84%)	17 (85%)	20 (83%)	0.8	19 (90%)	18 (78%)	0.3	15 (88%)	8 (73%)	14 (87.5%)	0.5
Treatment delivery	29 (65%)	14 (70%)	15 (62.5%)	0.6	18 (87.5%)	11 (48%)	< 0.01	14 (82%)	6 (54.5%)	9 (56%)	0.2
Follow-up	17 (39%)	10 (50%)	7 (29%)	0.16	9 (42.8%)	8 (43.7%)	0.58	8 (47%)	4 (36%)	5 (31%)	0.6
Hardware quality assessment	25 (57%)	13 (65%)	12 (50%)	0.69	14 (66.6%)	11 (48%)	0.2	12 (70.5%)	6 (54.5%)	7 (44%)	0.3
Research	1 (2%)	1 (5%)	0	0.2	1 (5%)	0	0.29	1 (6%)	0	0	0.4
Human resources	6 (14%)	3 (15%)	3 (12.5%)	0.8	2 (9.5%)	4 (17%)	0.48	2 (12%)	2 (18%)	2 (12.5)	0.8

In 71% (*n* = 34) of departments, results were commonly presented to the team, either to the middle management/executive team (*n* = 34) or to the complete team (*n* = 17). In 10 departments (21%), the results were presented in written form, as a conference or hearing in 21 departments (44%), a combination of written form and conferences were used in 3 departments. In 26 departments (54%), measures were defined to improve processes after analyses.

## Reactive risk analyses

Concerning critical incidents reporting, 23 institutions used the public authority reporting system for serious events in the last 12 months, of which 6 departments did not use other systems for less serious events (13%). A total of 28 used hospital-specific reporting systems such as CIRS (critical incident reporting system, 58%), while 14 participants used multiple systems (29%). None of the participants used international systems (ROSEIS or “safety in radiation oncology” [SAFRON]). In total, 34 institutions had made a report during the last year, six did not report, and eight did not reply to this question. Most departments reported 1–10 incidents in the last year (*n* = 29, 60%). Availability of a risk manager or risk committee did not have significant impact on the number of reports neither did risk rounds/m&m conferences or CIRS newsletters.

In ten departments (21%), a member of the physicists’ team was responsible for the workup of incidents or near-incidents. In four departments, the medical head of the department was responsible (8%); in 27 departments (56%), incidents were processed by a committee. No difference between different types of departments was seen. Root cause analyses were most common for evaluation (*n* = 13, 27%).

In most departments, incidents led to definition of measures for safety improvement in the last year (*n* = 31, 65%). In 85% (*n* = 41) of departments, information about incidents was presented to the team, either to the middle management/executive team (*n* = 41) or to the complete team (*n* = 27). Most common were conferences or hearings (*n* = 30, 63%). Some departments communicated in written form (*n* = 4) or used both oral and written form (*n* = 6).

## Discussion

To illustrate approaches for RM in different countries, the European ACCIRAD project that started in 2011 systematically assessed nationwide regularities on incident reporting and prospective risk management [30]. The authors reported on national RM requirements in 10 countries, yet in Germany, the implementation of national regularities for RM in RO took until 2018. As only few scientific papers on approaches of RM in Germany exist [4, 18, 34], our results present a considerable support for enhanced communication about the topic, for the definition of guidelines and teaching content as well as a base for future research.

## Prospective risk analyses

In all, 87% of departments reported having performed about at least one prospective risk analysis. Furthermore, half of the departments conducted more than one risk analysis, which correlates with availability of a risk manager or committee, so does the total number of subprocesses included. In contrast, 10% of departments did not conduct a risk analysis. Availability of a risk committee/manager is a key part of securing organizational structure for patient safety [23]. As expected, conduction of risk analyses was facilitated after nomination of dedicated staff members for these positions [16, 23]. Most departments adhered to common recommendations concerning methods and protagonists, e.g., involving an FMEA and interprofessional teams. FMEA was recommended in multiple publications about RM in RO [16, 35–37]; however, deviation in methods might bear certain advantages as described recently [18].

Most departments included planning, patient setup, and first radiation treatment in the analyses, while treatment routine was only assessed by two-third of institutions. Analysis of possible medication errors in general or during chemo-/immunotherapy were only reported by one institution in spite of their increasing role for patient safety [38, 39]. Available data identify the planning process as especially prone to severe errors [3]. Nevertheless, patient assessment, information transfer, and routine RT were also identified as very prone to events and near-events and should be included in risk analyses [3, 7, 9]. A possible reason for the difference in considering different process steps for analyses might be the fact that planning or setup/first treatment as subprocess are often conducted interprofessionally. This implies at least a four-eye-principle control of procedures and commonly discussions about possible improvements (peer reviews). During peer review of plans, possible safety concerns are discussed leading to plan modification in up to 66% of cases [19]. In contrast, patient assessment or routine RT are conducted by few staff members in shorter time [40] and are rarely presented in review rounds [23]. Especially in our cohort, 29% of departments reported that medical physicists were responsible for analyses which might enhance the effect of focusing on planning and setup. A similar effect was previously reported from other countries [41], underlining the fact that several subprocesses are commonly underrepresented in important peer-review-based quality control, even though it is recommended to pay as much attention to, for example, RT routine as to technical plan quality [42]. The limitation to 13 subprocesses to choose from in the questionnaire might not have fully covered all institution-specific workflows or nomenclatures, which impedes interpretation of the results. Nevertheless, it is conform with several recommendations for an institution to illustrate their own workflows and define specific subprocesses in a selectable extend before risk analyses [6, 8, 16].

## Reactive risk analyses

Use of incident-reporting systems is recommended by multiple societies and mandatory in Germany throughout healthcare institutions [43, 44]. The majority of departments in our study used incident reporting with a focus on hospital/institution specific reporting systems, e.g., CIRS [45]. This is in line with previous work showing that the use of internal reporting systems is most popular among RO staff [19, 46]. Most departments reported less than 10 incidents in the last year, and some did not even report at all. None of the participants used international reporting systems. Hesitation to report an incident or near-event is described by previous publications [19, 47] and underlines the need for change towards a more open failure culture (e.g. “just culture” [23]). Another reason for low reporting rates could be a lack of education about availability, handling, and most important benefit of reporting systems for patient safety [19, 47] and should be addressed in educational sessions. Implementation of a low-barrier institutional reporting-system for the RO department is recommended and similarly implemented in German nuclear medicine and radiology departments [48] or in other nations’ RO (e.g., PRISMA-RT in the Netherlands [49]). Since 2019, the German Department for Radiation Protection offers evaluation and recommendations in form of annually reports based on reported major incidents (Bedeutsame Vorkommnisse in der Medizin [BeVoMed]). Increasing benefit from the elaborated recommendations might encourage experts to aim for a generally more open reporting culture in the future. None of the “tools” of RM impacted the reporting rate. Most departments adhered to common recommendations and were able to define safety measures based on the analyses using recommended methods within an interprofessional team which was similarly described for prospective RM.

## Self-assessment and need for education

Most participants rated their knowledge about RM to be average or below. Most participants wished for more information by respective societies—offered via guidelines or special courses. Neither QM accreditation nor type of institution seemed to influence self-assessment. This is in line with results of a survey among American RO residents which describes low expertise regarding RM, although a program to improve skills on RM/QM existed for the RO curriculum [50]. Even though inclusion of QM and RM in medical education is recommended in Germany [51], current evaluation of RO residency training in Germany does not offer information on specific education on patient safety/RM issues so far [52, 53]. This might be included in further evaluation as this is a dedicated topic of international recommendations [54]. Division-specific education can be achieved by communicating existing RM processes. In our analyses, results of risk analyses—prospective and reactive—were mainly passed to the departments’ management levels. Especially regarding prospective analyses, only a minority of institutions communicated the results with all staff members (35%). As stated before, education about patient-safety issues shall be one key task of the management level (supported by the risk management committee) [22].

University hospitals reported more often about risk managers/committees, m&m conferences, and support by superior RM. However, neither prospective risk analyses nor incident reporting were performed more frequently in these institutions. It is especially noteworthy that self-assessment of knowledge on RM did not show different values for accredited departments. This could be caused by interpersonal differences in QM and RM knowledge, on the one hand, and the fact that the preparation for the accreditation process does not provide secure handling of RM tools to a satisfying extent, on the other hand, even though accreditation was previously described as an important component of patient safety [23, 31].

An advantage of larger institutions might be the increased accessibility of personnel resources, on the one hand, and support by implemented quality and risk management structures, on the other hand. Translation of recommendations, e.g., about certain tools of RM, might be facilitated. Nevertheless, implementation of a profound safety-culture including educational elements is possible in any RO department [51]. Departmental education should be facilitated by the societies focusing on national conditions. Leaned to existing recommendations [17], we expect updated guidelines from national societies and increasing focus on RM at national congresses and educational events [53].

## Limitations

Multiple institutions reported on tasks of RM being processed interprofessionally, so it is expected that answers represent impressions of interprofessional work. Nevertheless, especially for those departments where one person or profession is responsible for RM issues, answers might not completely represent balanced insight about the department’s viewpoints and knowledge about RM. For closer investigation of certain aspects which emerged as interesting from our survey, e.g., the role of accreditation, more detailed investigations are needed to obtain profound insight into the role of these points for development of a sustainable RM/patient safety structure.

A limitation of our study is that only 48 evaluable survey forms out of 294 RO departments in Germany were returned. We sent invitations for the survey with the aim to reach as many physicists as possible. Nevertheless, the absolute number of institutions is not completely clear. Assuming we reached all departments in Germany, the response rate would be 16%. As several departments of a region might be pooled under a common administration, participants of these could have answered the questionnaire once referring to practices in all subsumed departments. As the survey was designed to be anonymous, this bias cannot be excluded. A reason for a low response rate could be the lack of time during daily routine allowing staff to complete survey forms [40]. In addition, institutions without implemented processes of risk management might hesitate to take part in a survey about this topic. This may contribute to a low response rate, but also implies a bias of results, as our cohort might represent a rather experienced group in RM. Nevertheless, the return rate is comparable to other publications evaluating similar topics [50, 50, 55, 56].

## Conclusion

Our results reveal disparities in approaches for prospective and reactive RM in German RO departments, even though most departments adhered to current recommendations and regulations. Challenges in terms of implementation and realization of RM were identified and we showed that implementation of certain tools or protagonists such as risk managers can be of use in maintaining RM processes. We showed that the need for updated recommendations and education by technical societies exists throughout the entire institutional landscape.

## Recommendations

Based on our results and existing literature, the authors encourage all departments to increase efforts for education of their staff concerning risk management, including outcomes of in-house risk analyses. We suggest increasing the frequency of prospective risk analyses and to incorporate “routine processes”. Access to reporting systems for all personnel including use-briefing and definition of criteria for events should be provided. Existing examples for low-threshold reporting systems from RO worldwide or from other specialties could be adapted for German RO proposes on the institutional or cross-regional level. Future research can evaluate a broader spectrum of patient safety-related issues and safety culture in our field. Questions to address based on our results may be how communication and education concerning incidents, reporting, prospective analyses, and measures can be improved and how incident-learning can be transferred to maintaining changes in processes or how safety concepts can be assessed.

## Supplementary Information

Supplementary material: Survey about risk management – English transcript (original document in German)
