# Supplementary material for: Risk management patterns in radiation oncology—results of a national survey within the framework of the Patient Safety in German Radiation Oncology (PaSaGeRO) project
Source: Strahlenther Onkol. 2022 Aug 5;199(4):350–9. doi: 10.1007/s00066-022-01984-5 (PMC10033570; doi:10.1007/s00066-022-01984-5)
Supplement: Supplementary file 1 — Supplementary material: Survey about risk management – English transcript (original document in German) [file 66_2022_1984_MOESM1_ESM.docx]

**Supplementary material: Survey about risk management – English transcript (original document in German)**

**Affiliated to the following manuscript:**

Risk management patterns in radiation oncology – results of a national survey within the framework of the Patient Safety in German Radiation Oncology (PaSaGeRO) project in Germany

Andrea Baehr*, Daniel Hummel, Tobias Gauer, Michael Oertel, Christopher Kittel, Anastassia Löser, Manuel Todorovic, Cordula Petersen, Andreas Krüll, Markus Buchgeister

**^*Corresponding author. A. Baehr, Outpatient Center of the UKE GmbH, Department of Radiotherapy and Radiation Oncology, University Medical Center Hamburg-Eppendorf, Martinistr. 52, 20251 Hamburg, Germany andrea.baehr@uke.de^**

**Dear colleagues,**

**thank you for your interest in our survey, which will take approximately 10 minutes to answer.**

**Some questions allow multiple answers.**

**The results will be included in scientific evaluation and publication. Answers remain anonymous. With completing the questionnaire participants declare consent for including their answers in evaluation and publication.**

**(We would appreciate if you could provide us with in-house results of prospective risk analyses. More information is provided in our e-mail.)**

Part A: Prospective risk management

A1. Did you conduct prospective risk analyses in your department? (Analyses of risks, which might trigger critical incidents)

1. Don’t know
2. No
3. Yes, once
4. Yes, multiple times
5. Yes, regularly

A2. Which method was used to conduct the analysis in your department?

1. FMEA
2. Risk matrix
3. Bow tie analysis
4. Fault tree analysis
5. Don’t know
6. No answer
7. Other:

A3. Which statements apply to the conducted analysis?

1. It was conducted with an interprofessional team
2. It was conducted by medical physics’ staff only
3. An extern consultant was involved
4. An expert from the superior quality or risk management team was involved
5. None of the above mentioned applies
6. No answer
7. Other:

A4. How was the analysis conducted?

1. By a single person
2. By several persons in a conference
3. By several persons in several conferences
4. As a survey
5. As a survey using the Delphi-method
6. Don’t know
7. Other:

A5. Which subprocesses were included in the analysis? (Multiple answers)

1. patients‘ assessment
2. decision to treat and prescription
3. positioning
4. Imaging
5. volume definition
6. data transfer
7. planning
8. patient setup and first treatment
9. treatment delivery
10. follow-up
11. hardware quality assessment
12. research
13. human ressources
14. don’t know
15. no answer
16. other:

A6: Which modalities were included in the analysis? (Multiple answers)

1. Brachytherapy
2. Therapy with photons
3. Therapy with electrons
4. X-Ray-Therapy
5. Other:

A7: Did your department conclude measures relating to the analysis in the last 12 months? (Multiple answers)

1. No
2. Don’t know
3. Yes, less than five
4. Yes, five- ten
5. Yes, more than ten
6. Measures have been implemented partly or completely
7. Measures have not yet been implemented

A8. How were incidents and near-events announced among the department’s staff?

1. No announcement among the staff.
2. Among the team leaders in written form
3. Among the team leaders as hearing or conference
4. Among all personnel in written form
5. Among all personnel as hearing or conference
6. Don’t know
7. No answer
8. Other:

Part B: Analyses of incidents and near-events

B1: Which of the following reporting systems were used in your department in the last twelve months?

1. SAFRON (Safety in radiation oncology)
2. ROSEIS (radiation oncology safety education and information system)
3. Public authority reporting system (Meldung an die zuständige Behörde nach Strahlenschutzverordnung)
4. In-house reporting systems (e.g. critical incidents reporting system CIRS)
5. None
6. Don’t know
7. No answer
8. Other:

B2: How many reports were sent from your department in the last twelve months?

1. None
2. 1-10
3. 11-20
4. 21-50
5. More than 50
6. Don’t know

B3: How were incidents and near-events announced among the department’s staff?

1. No announcement among the staff.
2. Among the team leaders in written form
3. Among the team leaders as hearing or conference
4. Among all personnel in written form
5. Among all personnel as hearing or conference
6. Don’t know
7. No answer
8. Other:

B4. Who is responsible for work-up of incidents and near-events in your department?

1. Head of medical physics
2. Medical physicist
3. Head of medicine
4. Physician
5. Head of radiotherapists
6. Radiotherapist
7. Don’t know
8. No answer
9. Other:

A7: Did your department conclude measures relating to the analysis in the last 12 months? (Multiple answers)

1. No
2. Don’t know
3. Yes, less than 5
4. Yes, 5-10
5. Yes, more than 10
6. Measures have been implemented partly or completely
7. Measures have not yet been implemented

Part C: General information

C1. What kind of department is your current affiliation?

1. University hospital
2. University hospital with outpatients’ department
3. Hospital other than university hospital
4. Hospital other than university hospital with outpatients’ department
5. Private practice
6. Other:

C2: Which tools and protagonists are implemented in your department ?(Multiple answers)

1. Risk manager
2. Risk team
3. Support by an expert from the superior quality or risk management team
4. Support by extern consultants
5. Morbidity and mortality rounds
6. Other risk conferences
7. CIRS-Newsletter
8. Accreditation for quality management
9. Don’t know
10. Other:

C3. Would you wish for more support or more information about basics and methods in risk management? (Multiple answers)

1. No, we have sufficient information
2. Yes, as information or guidelines by medical or physical societies
3. Yes, as content of university education
4. Yes, as content of radiation oncology-specific continuous-education courses
5. Yes, other:

Thank you very much for your contribution.
